# Effects of guaifenesin, *N*-acetylcysteine, and ambroxol on MUC5AC and mucociliary transport in primary differentiated human tracheal-bronchial cells

**DOI:** 10.1186/1465-9921-13-98

**Published:** 2012-10-31

**Authors:** JeanClare Seagrave, Helmut H Albrecht, David B Hill, Duncan F Rogers, Gail Solomon

**Affiliations:** 1 Lovelace Respiratory Research Institute, 2425 Ridgecrest Dr SE, Albuquerque, NM 87108, USA; 2 Reckitt Benckiser, Parsippany, NJ, USA; 3 The University of North Carolina at Chapel Hill, Chapel Hill, NC, USA; 4 Airway Disease, National Heart & Lung Institute, Imperial College London, London, UK

**Keywords:** Expectorant, MUC5AC, Mucolytic, Mucus rheology, Respiratory infections

## Abstract

**Background:**

Therapeutic intervention in the pathophysiology of airway mucus hypersecretion is clinically important. Several types of drugs are available with different possible modes of action. We examined the effects of guaifenesin (GGE), *N*-acetylcysteine (NAC) and ambroxol (Amb) on differentiated human airway epithelial cells stimulated with IL-13 to produce additional MUC5AC.

**Methods:**

After IL-13 pre-treatment (3 days), the cultures were treated with GGE, NAC or Amb (10–300 μM) in the continued presence of IL-13. Cellular and secreted MUC5AC, mucociliary transport rates (MTR), mucus rheology at several time points, and the antioxidant capacity of the drugs were assessed.

**Results:**

IL-13 increased MUC5AC content (~25%) and secretion (~2-fold) and decreased MTR, but only slightly affected the G’ (elastic) or G” (viscous) moduli of the secretions. GGE significantly inhibited MUC5AC secretion and content in the IL-13-treated cells in a concentration-dependent manner (IC_50_s at 24 hr ~100 and 150 μM, respectively). NAC or Amb were less effective. All drugs increased MTR and decreased G’ and G” relative to IL-13 alone. Cell viability was not affected and only NAC exhibited antioxidant capacity.

**Conclusions:**

Thus, GGE effectively reduces cellular content and secretion of MUC5AC, increases MTR, and alters mucus rheology, and may therefore be useful in treating airway mucus hypersecretion and mucostasis in airway diseases.

## Background

Mucus in the airways is required to trap pathogens and inhaled particles for clearance via the mucociliary escalator towards the pharynx [1,2], where it and its trapped particles are either swallowed or expectorated. Exposure to irritants and pathogens causes leucocyte activation and inflammatory mediator release, which increase mucus production and enhance clearance of the inciting stimuli. However, the balance between mucus production and clearance [1] depends on optimal mucus quantities and hydration state, and periciliary fluid depth [3]. Airway bacterial or viral infections, asthma or chronic bronchitis can cause excessive mucus production and secretion. Combined with possible rheological changes, altered ciliary beating or uncoupling from the ciliary movement due to changes in liquid layer depth, these conditions can cause defective mucociliary clearance and airway mucus accumulation [1,4]. These factors lead, in turn, to coughing and subjective discomfort. In extreme cases, such as uncontrolled asthma, complete blockage of the airways with mucus can occur. There is, therefore, a need for improved therapeutic agents to improve mucociliary function under these pathophysiological conditions.

The major gel-forming mucins secreted by goblet cells and submucosal glands of the human upper respiratory tract are MUC5AC and MUC5B, respectively [5,6]. MUC5AC is considered a biomarker for airway goblet cells [7] and is widely used for studying goblet cell metaplasia *in vivo* and *in vitro*.

Pharmacological approaches for relieving mucus hypersecretion currently include several classes of agents [1]. Classic mucolytic drugs such as *N*-acetylcysteine (NAC) decrease the viscoelastic properties of mucus by reducing disulfide bonds. In contrast, expectorants change mucus consistency and make coughing more productive, mucokinetics improve transportability, and mucoregulators suppress mucus secretion.

We previously investigated the effects of guaifenesin ([3-(2-methoxyphenoxy)-1,2-propanediol], also called glyceryl guaiacolate ether, GGE) *in vitro*, using primary human airway epithelial cells differentiated by air-liquid interface culture [8]. These cultures are a complex, organotypic human airway model, containing the major cell types: basal, ciliated, non-ciliated and goblet cells [9]. Using this system, we demonstrated that GGE reduced the cellular content and secretion of MUC5AC, altered the viscoelastic properties of the secretions, and improved mucociliary transport rates (MTR) [8]. Here, we extend these observations to conditions in which MUC5AC secretion was increased by culturing with IL-13 for 3 days prior to analysis [10]. We compared the effects of the expectorant, GGE, with a mucolytic (NAC), and another mucoactive/expectorant agent, ambroxol (Amb), a drug reported to have anti-inflammatory, antioxidant and anaesthetic effects [11]. Previous studies have suggested that IL-13 stimulation increases MUC5AC production (but not MUC5B) [12]. We hypothesised that these drugs would affect MUC5AC content and secretion and improve mucus rheology, thereby improving MTR, in these stimulated cells.

## Materials and methods

### Antioxidant capacity assay

Trolox equivalent antioxidant capacity was measured essentially as previously described [13]. Dilutions of the mucoactive agents and Trolox, a water-soluble vitamin E analogue used as a standard, were prepared in Dulbecco’s phosphate-buffered saline (DPBS), previously sparged with nitrogen, and incubated with previously heated 0.23 mM 2,2'-azinobis-(-3 ethylbenzothiazoline-6-sulphonate)/2.3 mM 2,2'-azobis-(-2-amidinopropane). The optical density (734 nm) was read using a VersaMax (Molecular Devices, Inc., Sunnyvale, CA USA) plate reader 5 minutes after initiating the reaction.

### Cell cultures and exposures

Cultures of primary human airway epithelial cells, differentiated at air-liquid interface for 2 weeks on 1 cm^2^ transwells for the MUC5AC content and secretion assays or on 4.2 cm^2^ transwells for the mucociliary transport and rheology assays (EpiAirway), were purchased from MatTek (Ashland, MA, USA) and maintained according to the supplier’s recommendations. A single donor was used for all experiments. Except where noted, IL-13 (Lab Vision, Thermo Fisher Scientific Inc., Kalamazoo, MI, USA) was added at 1 ng/mL to the basolateral compartment for 3 days, replaced daily, prior to treatment with the mucoactive agents: GGE (provided by Reckitt Benckiser, Parsippany, NJ, USA), NAC or Amb (Sigma-Aldrich, St. Louis, MO, USA) [10]. For assessment of MUC5AC content and secretion, the apical surfaces were washed twice with DPBS immediately before treatment with the mucoactive agents. To ensure that sufficient mucus was present for the analysis of mucociliary transport and mucus rheology, cultures for these experiments were washed 24 hr before treatment. The mucoactive agents, diluted to 10, 30, 100 and 300 μM in culture medium containing 1 ng/mL IL-13 were provided basolaterally. Cultures treated with IL-13 alone served as the “no drug” control, and an additional set received neither IL-13 nor drug. Each endpoint evaluated the responses from a set of individual cultures: the number of cultures is reported for each assay.

### Cell viability and cellular levels and release of MUC5AC

For each analysis time point, quadruplicate cultures were treated as described above. At the designated times, the apical surfaces were gently washed twice with 100 μL of DPBS containing 0.1 mM dithiothreitol (DTT). The washes were centrifuged and the supernatants were frozen for subsequent ELISA analysis of MUC5AC content. Viability was assessed using the Water Soluble Tetrazolium (WST-1) assay (Roche Applied Science, Indianapolis, IN, USA). The cells were then lysed with 200 μL of 1% Triton X-100, 2 mM EDTA and 10 μM Pefabloc (Roche Applied Science) in DPBS. Following centrifugation, the supernatants were frozen for subsequent ELISA analysis.

### MUC5AC ELISA

The ELISA followed previously described procedures [8], using anti-MUC5AC antibody (45M1; Thermo Fisher), peroxidase-conjugated secondary antibody, and high-sensitivity tetramethylbenzidine development (Thermo Fisher). The washes were diluted 1:4 and the lysates were diluted 1:10 in DPBS for analysis. Standards consisting of a pooled sample of EpiAirway secretions were prepared in DPBS with 0.025 mM DTT for analysis of the secreted MUC5AC, and in DPBS without DTT for analysis of the lysate MUC5AC content, to account for the non-linear relationship between concentration and signal intensity and differences in antibody binding in the presence of low levels of DTT. The standard curve was fit to a 4-parameter regression, and the samples were within the mid-range of this curve. The data are reported as concentrations relative to the undiluted pooled sample. None of the mucoactive agents interfered with the assay.

### Mucociliary transport

**Figure 1:**
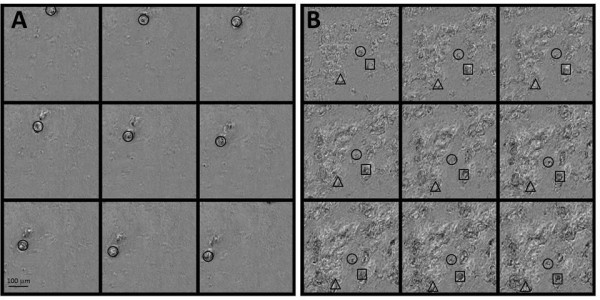
**Examples of “individual particles” or “mucus sheets”.** The video files were processed using CISMM software ( http://cismm.cs.unc.edu/downloads/?dl_cat=3) to remove the invariant background, thus emphasizing the moving material, and every tenth frame is shown. Panel **A** shows an example of a “single particle,” indicated by a circle, although a second particle is present. Panel **B** shows an example of a “mucus sheet” where many elements are present, three of which are indicated as a circle, a square, and a triangle, that move together.

A separate set of EpiAirway cultures, similarly pre-treated with IL-13, were treated in sextuplicate with the mucoactive agents at either 30 or 100 μM. After 3, 8 or 24 hr, videomicroscopy data were collected. In most cases, three fields, selected on the basis of containing endogenous moving material, were imaged for each of four cultures. Post-imaging processing to assess the rate of movement of endogenous debris on the surface of the cultures was performed from the video, attempting to track at least five particles for each microscopic field. In some cases there was insufficient material to meet these targets. The images were subjectively classified as “individual particles” or “mucus sheets”, where mucus sheets were considered to be larger masses of material moving synchronously across the field. Examples are provided in Figure 1. After the 8-hr time point, undiluted apical secretions were harvested from 2 cultures per exposure condition and frozen for subsequent micro-parallel plate rheology. The apical secretions were similarly collected from the remaining four cultures after the 24-hr imaging.

### Rheology

The rheological properties of the mucus samples harvested from the cell cultures were ascertained by performing amplitude sweep experiments on a Bohlin Gemini Rheometer (Malvern Instruments, Worcestershire, UK), with a 20 mm diameter parallel plate set at a gap thickness of 50 μm. All experiments were performed over a stress range of 0.025–50 Pa and at a frequency of 1 Hz (intermediate frequency between those associated with tidal breathing and mucociliary clearance). All analyses were performed at 23°C to minimise sample dehydration [14]. For each sample, we report data from the linear regimes, established through methods similar to those of Vasquez et al. [15].

### Statistical analysis

Statistical analysis of the effects of the test materials on viability and mucus secretion and production were performed using ANOVA with Dunnett’s post-test, comparing all samples against the IL-13-only control sample at each time using GraphPad Prism software (version 5.04). In addition, a trend test evaluating the effect of each drug as a function of concentration, including the IL-13-only cultures as the zero drug concentration, was performed. The effective concentration for 50% inhibition (IC_50_) of the cellular MUC5AC content and secretion was determined by fitting an exponential decay function using Prism.

## Results

### Antioxidant capacity

**Figure 2:**
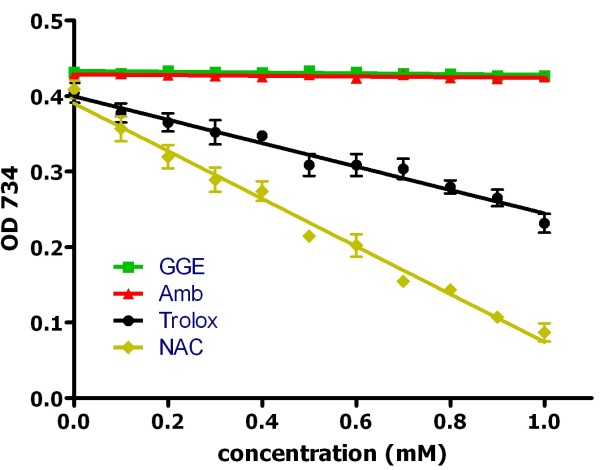
**Antioxidant capacity of guaifenesin (GGE), *N*-acetylcysteine (NAC) and ambroxol (Amb).** The ability of the three mucoactive agents to scavenge the free radical/colorimetric indicator as a function of concentration was compared with Trolox (a water soluble vitamin E analogue). NAC was approximately twice as effective (slope -0.316) as Trolox (slope -0.155), but the effects of GGE and Amb were minimal (slopes of 0.032 and 0.025, respectively).

NAC is known to alter mucus viscosity by reducing the intramolecular disulphide bridges. We therefore investigated whether either of the other agents had redox-active properties. NAC had approximately twice the antioxidant capacity of Trolox, with a slope of -0.316 vs. -0.155 (Figure 2). Neither GGE nor Amb had any evident antioxidant capacity, with slopes of 0.032 and 0.025, respectively.

### Viability

**Figure 3:**
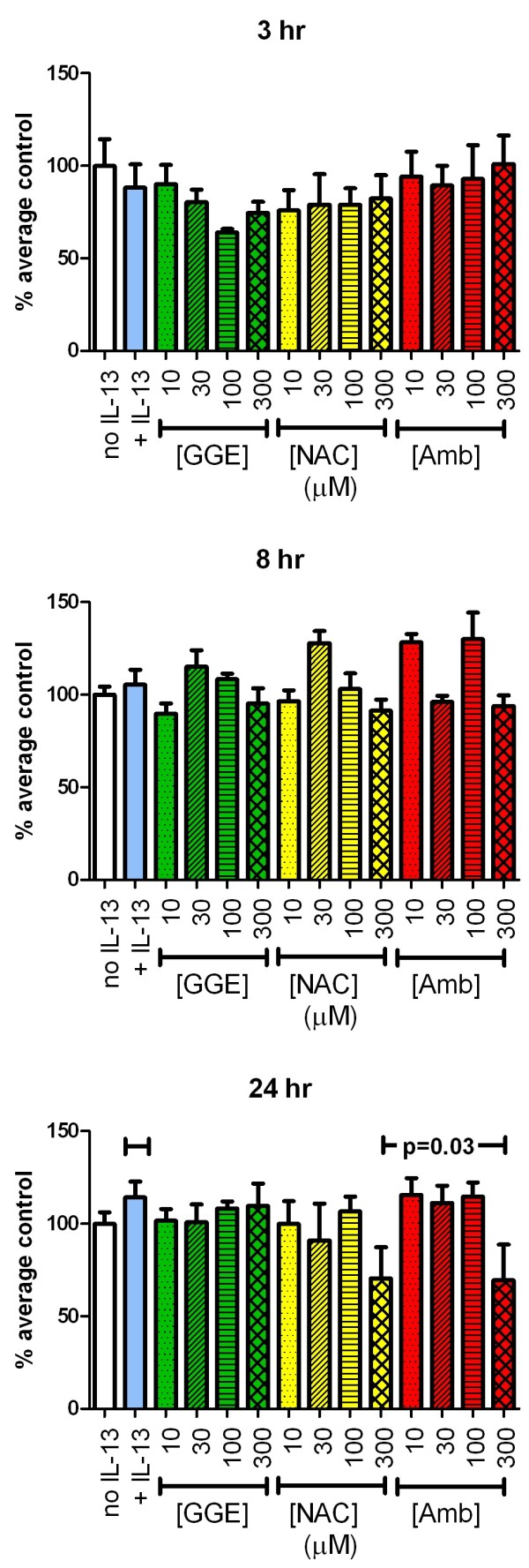
**Effects of guaifenesin (GGE), *N*-acetylcysteine (NAC) and ambroxol (Amb) on cell viability.** Viability after 3, 8 or 24 hr of incubation with the concentrations of the drugs as indicated was assessed using the Water Soluble Tetrazolium (WST-1) assay. The horizontal line indicates the p-value for a statistically significant trend for ambroxol at 24 hr, when the IL-13-only cultures were included as the zero drug concentration as indicated by a short line over the IL-13-only bar at the same height. (The trend test does not include the comparator negative control culture, which was not treated with IL-13).

It is possible that a decrease in secretion or altered mucociliary transport could result from toxicity of the drugs tested. In order to confirm the lack of toxicity, we assessed viability of the cells using the WST assay. None of the experimental interventions significantly altered cell viability at any time point studied based on the ANOVA analysis comparing all individual data to the IL-13-alone control (Figure 3). However, there was a weak, but statistically significant trend, for Amb at the 24 hr time point.

### MUC5AC secretion and content

**Figure 4:**
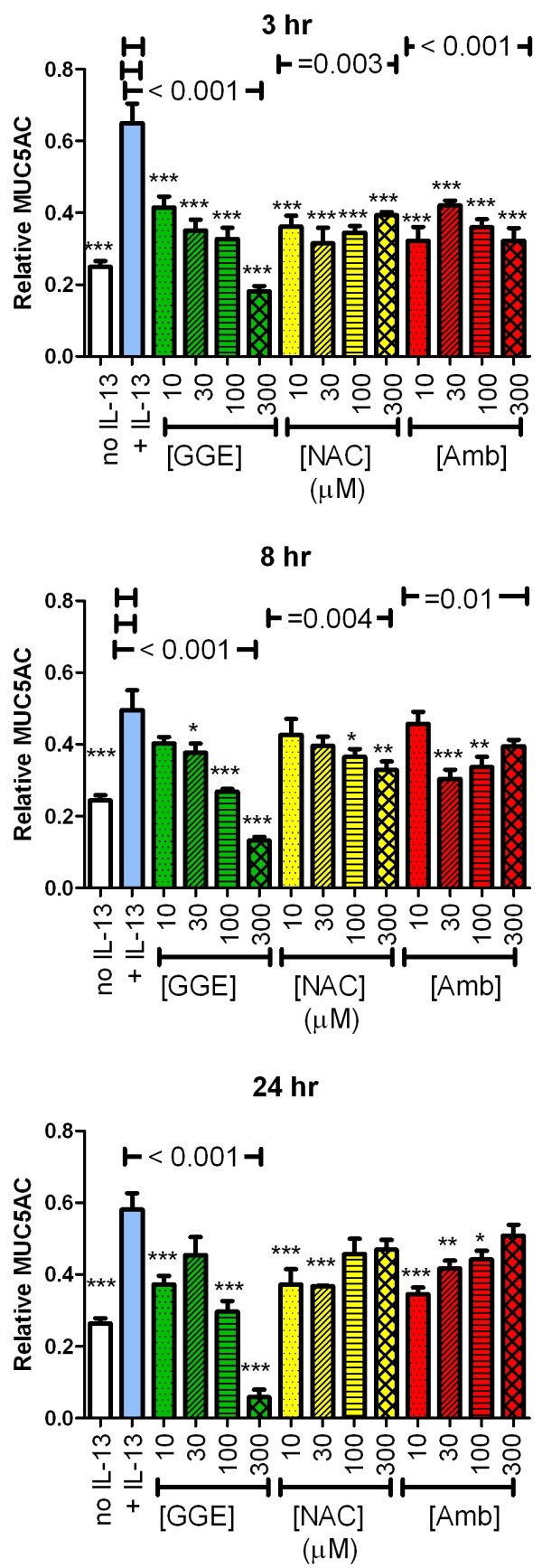
**Effect of guaifenesin (GGE), *N*-acetylcysteine (NAC) or ambroxol (Amb) on secretion of MUC5AC.** The MUC5AC concentrations in two washes of the apical surfaces of quadruplicate cultures for each condition were analysed by ELISA. All samples and standards included 0.025 mM dithiothreitol (DTT), resulting from the 1:4 dilution of the samples collected in 0.1 mM DTT. The results from the two washes were combined and are reported as the concentration relative to the standard curve, generated from dilutions of a pooled sample of washes collected from other samples. Separate cultures were analysed at the three time points. *, ** and *** indicate p < 0.05, 0.01 and 0.005, respectively, vs. IL-13-only group (ANOVA with Dunnett’s post-test). Horizontal lines above the groups indicate significant trend tests (all include the IL-13-only, zero drug, condition as indicated by the short line over this bar at the same height).

**Figure 5:**
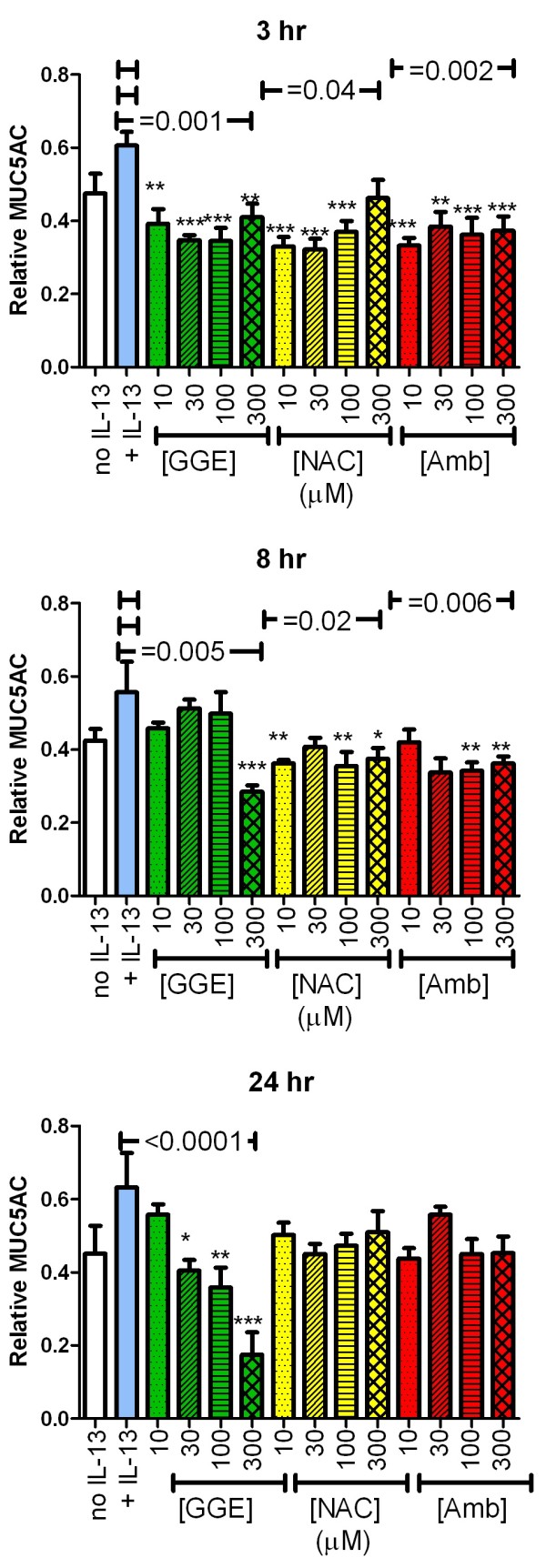
**Effect of guaifenesin (GGE), *N*-acetylcysteine (NAC) or ambroxol (Amb) on cellular MUC5AC content.** The MUC5AC content in lysates of quadruplicate cultures for each condition were analysed by ELISA. No dithiothreitol was used in this ELISA because the lysis buffer did not contain this reducing agent. The results are reported as concentration relative to the standard curve, generated from dilutions of a pooled sample of washes collected from other samples. Separate cultures were analysed at the three time points. *, ** and *** indicate p < 0.05, 0.01 and 0.005, respectively, vs. the IL-13-only group (ANOVA with Dunnett’s post-test). Horizontal lines above the groups indicate significant trend tests (all include the IL-13-alone, zero drug, condition as indicated by the short line over this bar at the same height).

Based on our previous evidence that GGE affected MUC5AC production and secretion, we examined the effects of all three drugs on this parameter in the IL-13-stimulated cells. IL-13 pre-treatment increased MUC5AC secretion in the pooled washes 2-fold (Figure 4) and cellular content by 25% (Figure 5). GGE significantly inhibited IL-13-induced MUC5AC secretion in a concentration-dependent manner at 3, 8 and 24 hr, with IC_50_ values (95% confidence intervals, CI) of 137 (73–102), 130 (98–190) and 96 (85–109) μM, respectively (Figure 4). At 8 and 24 hr, 300 μM GGE reduced secretion below the levels of non-IL-13-treated cells, consistent with our previous observations [8]. NAC and Amb both significantly reduced the levels of the secreted MUC5AC for some concentrations. However, the effect was not consistently concentration-dependent and no clear pattern was observed. Inhibition by 300 μM Amb or NAC was much less than at the same concentration of GGE.

Similar to the effects on secretion, GGE decreased the cellular content of MUC5AC in IL-13-treated cultures at all time points, with an IC_50_ value at 24 hr of 150 (95% CI: 95–330) μM (Figure 5); the cellular content of MUC5AC in 300 μM GGE-treated cultures was less than that in the non-IL-13-treated cells. Amb and NAC also decreased the cellular content of MUC5AC in IL-13-treated cultures at 3 and 8 hr, but not at 24 hr (Figure 5). The inhibition at 3 and 8 hr was not obviously concentration-dependent.

### Mucociliary transport

**Figure 6:**
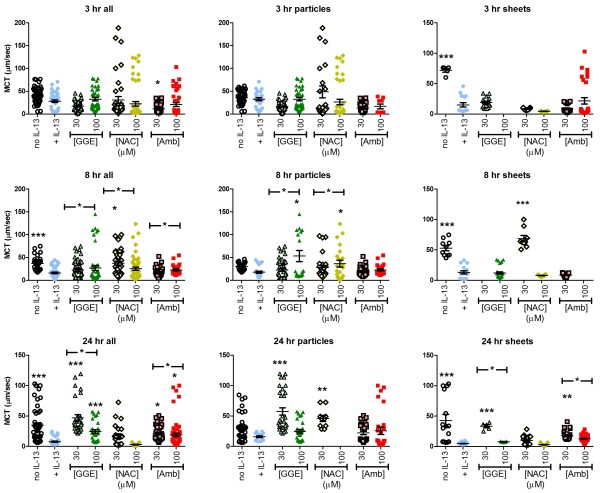
**Effect of guaifenesin (GGE), *N*-acetylcysteine (NAC) or ambroxol (Amb) on mucociliary transport rates.** Individual particle rates for endogenous particles on the surface of human primary lung epithelial cell cultures are indicated by symbols. Lighter coloured symbols with black borders indicate the 30 μM concentration of the drugs, while darker solid symbols indicate the 100 μM concentration of the drugs. Target data collection was for at least 5 moving elements from each of three fields on each of 4 cultures. In some cases, insufficient numbers of particles or fields with particles were found. Panels on the left side indicate the combined data from elements subjectively designated as individual particles or those associated with mucus sheets. The middle panels show only those designated as individual particles and the panels on the right show only those associated with mucus sheets. *, ** and *** indicate p < 0.05, 0.01 and 0.005, respectively, vs. the IL-13-only control (ANOVA with Dunnett’s post-test). Horizontal lines above the groups indicate trend tests with p values < 0.05 (all include the IL-13-alone, zero drug, condition as indicated by the short line over this bar at the same height).

A key factor in the clearance of mucus from the airways is MTR. We examined the effects of IL-13 and the effects of the drugs on MTR in the IL-13 stimulated cells. Figure 1 shows examples of processed video files of “individual particles” or “mucus sheets”. IL-13 reduced MTR for combined particles+mucus sheets (denoted “all” in the figure), and for mucus sheets alone, significant for all except the 3hr combined samples (Figure 6). The effects at 3, 8 and 24 hr for mucus sheets were 80, 75 and 89% reductions, respectively. These decreases in MTR over the 24 hr observation period followed 3 days of preincubation with IL-13.

At the 3 hr time point, none of the drugs had any significant effect on the IL-13-induced reduction in MTR (Figure 6); this was associated with marked variability in response in some groups (e.g. 30 μM NAC, individual particles). By 8 hr, MTR was 40 and 66% greater in cultures treated with 30 and 100 μM GGE, respectively, relative to the IL-13-only cultures; significant for 100 μM, and with a marked biphasic effect on individual particles. The lack of effect of 30 μM GGE on “all” MTR was associated with the absence of mucus sheets in this group. There was also a > 2-fold increase in MTR in cultures treated with 30 μM NAC, driven by a marked effect on elements within sheets. At 100 μM NAC, there was also a 2-fold increase in rate for individual particles, but a decrease for those present in the mucus sheets. Neither concentration of 100 μM Amb significantly altered MTR, although there was a weak trend towards an increase. At 24 hr, 30 μM GGE increased MTR of the combined material over 6-fold relative to the IL-13-only, ~4-fold for individual particles and ~7-fold for mucus sheets (Figure 6). The effect of 100 μM GGE (3-fold increase) was only significant for the combined data. NAC at 30 μM significantly increased MTR (2-fold) for both individual particles and mucus sheets at 24 hr. In contrast, for cultures treated for 24 hr with 100 μM NAC (Figure 6), only one field with any particles was found in four cultures examined, and this field had a mucus sheet with very low mobility. Amb at 30 and 100 μM significantly increased MTR for combined material (~2.5-fold) and sheets (~3.5- and ~2.5-fold), respectively, but the effects on individual particles (40 and 60% increases) were not significant. Multivariate analysis indicated significant increases in MTR at 100 μM GGE and both concentrations of NAC relative to the IL-13 group, irrespective of time or particle type. The effects of Amb were not significant. There were also significant effects of particle type, irrespective of time or treatment: particles within mucus sheets had lower MTR than individual particles.

### Rheology

**Figure 7:**
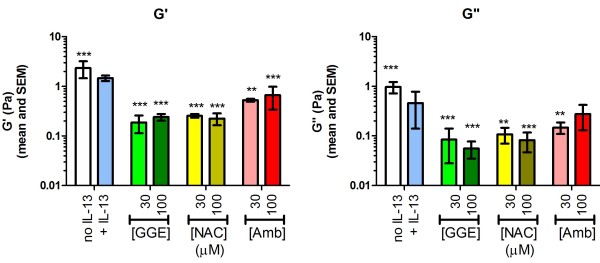
**Effects of guaifenesin (GGE), *N*-acetylcysteine (NAC) or ambroxol (Amb) on rheological parameters.** G’ (elasticity) and G” (viscosity) of the secretions from human primary lung epithelial cell cultures at 24 hr are shown. Elasticity and viscosity of collected, undiluted, apical secretions were evaluated using a 20 mm parallel plate rheometer at a 50 μM gap size. ** and *** indicate p < 0.01 and 0.005, respectively, vs. the IL-13-only control (ANOVA with Dunnett’s post-test).

Increases in mucus viscosity and elasticity contribute to mucostasis and its pathophysiological consequences. Alterations in these parameters by sulfhydryl-reactive agents such as NAC are thought to be the primary mechanism for the improved mucus clearance for these agents. We therefore examined the effects of the three drugs on mucus rheology. IL-13 slightly but significantly decreased elasticity (G’) and viscosity (G”) moduli at 24 hr (Figure 7). However, both GGE and NAC at either concentration decreased both parameters by approximately one order of magnitude. Amb was less effective, significantly decreasing these parameters by 60–70% relative to the IL-13 group. Few of the samples collected at 8 hr contained sufficient material for analysis. Where sample quantities were sufficient (control, IL-13-treated, 30 μM GGE, 100 μM NAC and 100 μM Amb), the results were generally consistent with the 24 hr results: GGE and NAC reduced both G’ and G”, whereas Amb did not affect G’ and increased G”.

## Discussion

In the present study, we found that IL-13 increased the cellular content and secretion of MUC5AC and decreased mucociliary transport. Interestingly, the cytokine caused small decreases in both G’ and G”, which were statistically significant. However, the changes were less than 2-fold, and the biological significance of this effect is probably minimal. None of the three mucoactive agents were cytotoxic at the concentrations tested, but they differed substantially in their properties. Only NAC potently scavenged free radicals, while only GGE had a concentration-dependent capacity to decrease the cellular content and secretion of MUC5AC. In contrast, all three drugs affected the rheology of the secretions.

Mucolytics generally decrease mucus viscosity by reducing the dicysteine bridges that contribute to the rigidity of the mucins [16]. In the present study, NAC had relatively small effects on the cellular content or secretion of MUC5AC. However, it significantly decreased the viscosity and elasticity parameters of the secretions. Interestingly, NAC also appeared to reduce the total quantity of endogenous debris and, in particular, the fraction present as part of the mucus sheets. This aspect of the regulation of mucociliary function may merit additional study. Furthermore, 100 μM NAC decreased MTR for the particles associated with mucus sheets to below those of the IL-13-only cultures at all time points. It is possible this resulted from strong effects on viscosity that disrupted effective coupling between the mucus and the beating cilia.

Amb is used clinically to improve cough and discomfort from excessive mucus accumulation. Its mode of action is not fully understood, but may include antioxidant, anti-inflammatory or anaesthetic modes of action [11]. It also may increase surfactant production [1] and alkaline phosphatase secretion (which is associated with surfactant, but also reduces the effects of lipopolysaccharide) [17], and alter ion transport [18]. In our studies, Amb suppressed MUC5AC secretion and decreased MUC5AC cellular content slightly, but the effect was not concentration-dependent. Although this agent also altered the rheological parameters, the effect was substantially smaller than that of either NAC or GGE.

Several hypotheses for the mechanisms of action for GGE have been proposed. One suggested mechanism, the neurogenic hypothesis, involves stimulation of receptors in the stomach, resulting in vagal stimulation of respiratory tract fluid secretion, specifically cholinergic parasympathetic reflexes activating submucosal gland secretions [19]. There is also evidence for direct effects on mucus adhesiveness and surface tension [20-22], and on cough sensitivity [23]. Significant effects on mucociliary clearance have also been demonstrated in chronic bronchitis patients but not in healthy controls [20]. In contrast, no direct effects on mucociliary clearance measured as saccharine transport time *in vivo* or ciliary beat frequency in nasal epithelial cell samples isolated from GGE-treated healthy volunteers were observed, although sampling artefacts in the isolation of the cells might have affected the results [24]. In the present study, in contrast to the other agents tested, GGE produced substantial concentration-dependent decreases in both cellular MUC5AC content and secretion in the IL-13-stimulated cultures, similar to our previously reported results in unstimulated cells [8]. The effects on MUC5AC content and secretion were paralleled by increases in MTR and significant decreases in both elasticity and viscosity parameters.

Added value in our study is provided by the correlations among MUC5AC content and secretion, MTR and rheology, which support the concept that the drugs affect selected parameters of the mucociliary transport system. In addition, the use of endogenous materials to track the MTR is an advantage. This method avoids potential dilution of the secretions and alterations in rheological properties that result when a suspension of exogenous particles is added to the surfaces of the cultures. This study also has some limitations. While the differentiated primary human cell cultures are more realistic models of the airway than cell lines or conventionally cultured primary cells, there is no actual clearance of the secretions, and the cultures must be washed at intervals. For this reason, the mucus levels vary from very little immediately after washing, to potentially large, depending on when the last washes occurred. We attempted to standardise these procedures, but in order to ensure sufficient mucus for the rheology measurements, the cultures for the MTR and rheology measurements included mucus produced and secreted during the 24 hr preceding drug treatment, while the cultures used to assess MUC5AC content and secretions were washed immediately prior to drug treatment. We did not attempt to measure ciliary beat frequency or periciliary fluid levels. In addition, the use of human lung cells precludes evaluation of effects mediated by other organs, such as the neurogenic mechanism postulated for the early effects of GGE [19], which cannot be modelled in lung cell cultures. Metabolism by hepatic enzymes is also not included in the present model, and thus the time course for *in vivo* responses may differ from those observed *in vitro*. Finally, the contributions of mucins other than MUC5AC were not addressed in this study. These cultures exhibit modest numbers of MUC5AC-producing goblet cells [5], which are increased by IL-13 treatment [10], but not fully differentiated submucosal glands, the major source of MUC5B [6]. However, MUC5B has been shown to be the major mucin in these cultures [25]. It is, therefore, possible that the changes in mucus properties are due to the presence of MUC5B in the secretions. Future studies will address this possibility directly.

## Conclusions

In summary, this study indicates that GGE had a strong inhibitory effect on the production of MUC5AC in IL-13-stimulated cultures, which correlated with increased MTR and reduced viscosity and elasticity of the secretions. These effects were unlikely to have been the result of reduction of dicysteine bridges, given its lack of antioxidant capacity. In contrast, NAC did not significantly affect the cellular content or secretion of MUC5AC and had a biphasic effect on MTR (increased at 30 μM but suppressed at 100 μM, particularly for those particles associated with the mucus sheets). As expected, NAC reduced the rheological parameters. Amb, like NAC, had no significant effect on the cellular content or secretion of MUC5AC, but modestly increased MTR and reduced elasticity and viscosity. Thus, we conclude that in human differentiated airway epithelial cells under conditions mimicking an inflammatory response, GGE reduces MUC5AC production, increases MTR, and decreases mucus viscosity and elasticity. These results support the use of this drug for hypersecretory conditions of the airways, including bacterial or viral infections and chronic bronchitis.

## Abbreviations

Amb: Ambroxol; ANOVA: Analysis of Variance; DPBS: Dulbecco’s phosphate buffered saline; DTT: Dithiothreitol; EDTA: Ethylenediaminetetraacetic acid; ELISA: Enzyme-linked immunosorbant assay; G’: Elastic modulus; G”: Viscosity modulus; GGE: Guaifenesin; IC50: Concentration for 50% inhibition; IL-13: Interleukin 13; MTR: Mucociliary transport rate; NAC: *N*-acetylcysteine; WST-1: Water Soluble Tetrazolium.

## Competing interests

JS has received research funds from Reckitt Benckiser. HHA is a consultant to Reckitt Benckiser and is the co-author of a Mucinex (sustained-release guaifenesin) patent. DBH has received research funds and consultancy payments from Reckitt Benckiser. DFR has received consultancy payments from Reckitt Benckiser. GS is an employee of Reckitt Benckiser and is also a co-author of a Mucinex (sustained-release guaifenfesin) patent.

## Authors’ contributions

JS contributed to the experimental design, coordinated the study, performed the MUC5AC secretion and mucociliary transport analyses, and drafted the manuscript. HHA and GS conceived the study and contributed to experimental design. DBH performed the rheology analyses. DR provided guidance on experimental design and contributed to the interpretation. All authors read and approved the final manuscript.
